# Rosai and Dorfman Disease with Pleural Involvement: Case Report

**DOI:** 10.1100/tsw.2008.97

**Published:** 2008-08-31

**Authors:** Jouda Cherif, Sonia Toujani, Nadia Mehiri, Bechir Louzir, Nidham Kchir, Majed Beji

**Affiliations:** ^1^ Service de Pneumologie Allergologie, Centre Hospitalo-Universitaire La Rabta, 1006 Bab Saadoun Tunis, Tunisie; ^2^ Service d'Anatomo-Pathologie, Centre Hospitalo-Universitaire La Rabta, 1006 Bab Saadoun Tunis, Tunisie

**Keywords:** Rosai Dorfman, sinus histiocytosis, lower respiratory tract involvement

## Abstract

Sinus histiocytosis with massive lymphadenopathy (SHLM) disease is considered to be an indolent and self-limiting pathology. However, severe morbidity and mortality have been attributed to complications of SHLM. Lower respiratory tract involvement is rarely reported and is often unfavorable, and carries a particularly grave prognosis. A case of SHLM is reported, in which the patient had lower respiratory and pleural involvement.

## INTRODUCTION

Sinus histiocytosis with massive lymphadenopathy (SHLM) was identified and established as a distinct clinicopathologic entity following the reports of Rosai and Dorfman[1]. It was initially characterized as a nodal-based disease process. Since that time, it has been well established that SHLM may affect a variety of extranodal sites. A review of the cases from the registry of SHLM reported that 43% of patients with SHLM had at least one site of extranodal involvement. Extranodal SHLM can occur independently of lymph node involvement. The most common clinical manifestation of this disease is bilateral, nontender, painless, cervical lymphadenopathy. Lower respiratory tract involvement was rarely reported in the literature. We report a case of SHLM with lower respiratory tract and pleural involvement.

## CASE REPORT

A 58-year-old, Caucasian man presented in 1995 with a 4-month history of bilateral cervical adenopathy and 2 months of progressive dyspnea, coughs, and left chest pain. Physical examination on admission revealed massive lymphadenopathy in the anterior and posterior cervical chains bilaterally and the left inguinal region, in addition to xanthomatous-like skin lesions. The lung examination was normal. Laboratory investigations showed an erythrocyte sedimentation rate at 105 mm/h and normal white blood cell count. Chest X-ray (Fig. 1) showed a nodular infiltration of the lower right lung field in addition to pleural effusion. Tuberculin skin reaction was negative. Sputum showed no acid-fast bacilli on Ziehel-Neelson stain and cultures for mycobacteria were negative. Human immunodeficiency virus and

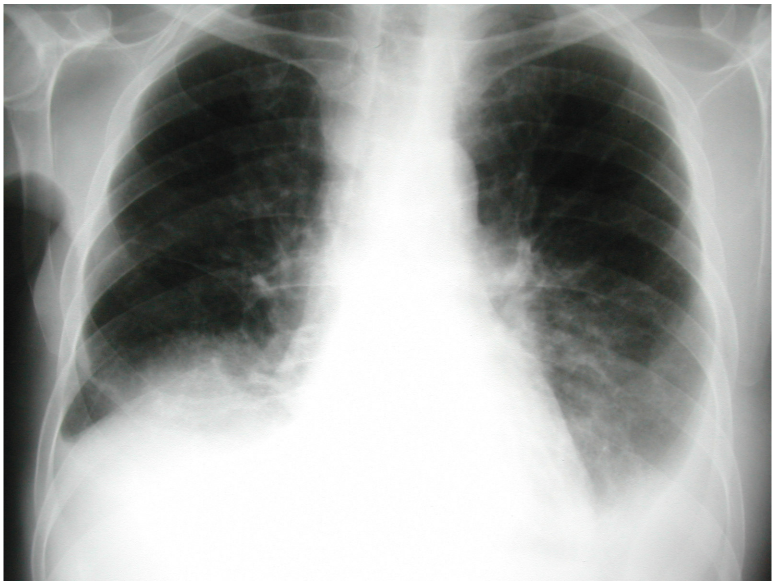

**FIGURE 1**. Chest X-ray.

toxoplasmosis serologies were negative. A cervical lymph node biopsy was performed, which showed capsular fibrosis and dilatation of subcapsular and medullary sinuses that were filled with active, but benign appearing, histiocytes that containing cellular debris, erythrocytes, and numerous lymphocytes expressing CD68 and S100 protein. A bronchial fibroscopy was performed, which showed numerous granular-appearing polypoid tissues on the wall of the main bronchus. Neither of these tumefactions compromised the airway. Bronchial, transbronchial, and skin biopsies showed histological appearances similar to the node biopsy, compatible with SHLM.

Thoracoscopy followed by pleural biopsy of nodular and inflammatory spaces showed exactly the same histological aspect of the cervical lymph node (Fig. 2).

At follow-up after presentation, the patient reported a progressive worsening of his symptoms and died 4 months later. Postmortem examination was not performed.

## DISCUSSION

Since the original descriptions of SHML, the extent of the extranodal manifestations of this condition has been well recognized. The etiology remains unknown, but it is thought to be a disorder of immune regulation or response to a presumed infectious agent[1].

Irrespective of the site of occurrence, the histopathologic appearance of SHLM is essentially similar. The hallmark is the tendency for the infiltrate to recapitulate lymph node architecture. This has been referred to as a “sinusal” pattern in which the clustering of lymphocytes stimulates the appearance of germinal centers with surrounding cellular proliferation and dilated sinuses. More fibrosis, fewer typical histiocytes, and a lesser degree of lymphophagocytosis are the minor histological differences between nodal and extranodal SHLM[2].

Rosai-Dorfman Disease (RDD) involving the thorax is rare and most often manifests as pulmonary disease, which accounts for only 2% of RDD cases[4]. The patients who develop pulmonary RDD are young (average age, 14 years), and have involvement of lymph nodes and other extranodal sites (nasal cavity, paranasal sinuses). The tracheobronchial tree is infiltrated most commonly and intraluminal polypoid growth of RDD may produce airway obstruction. Diffuse interstitial or air space involvement occurs less frequently; however, when there is extensive parenchymal involvement, the pleura may be involved[3].

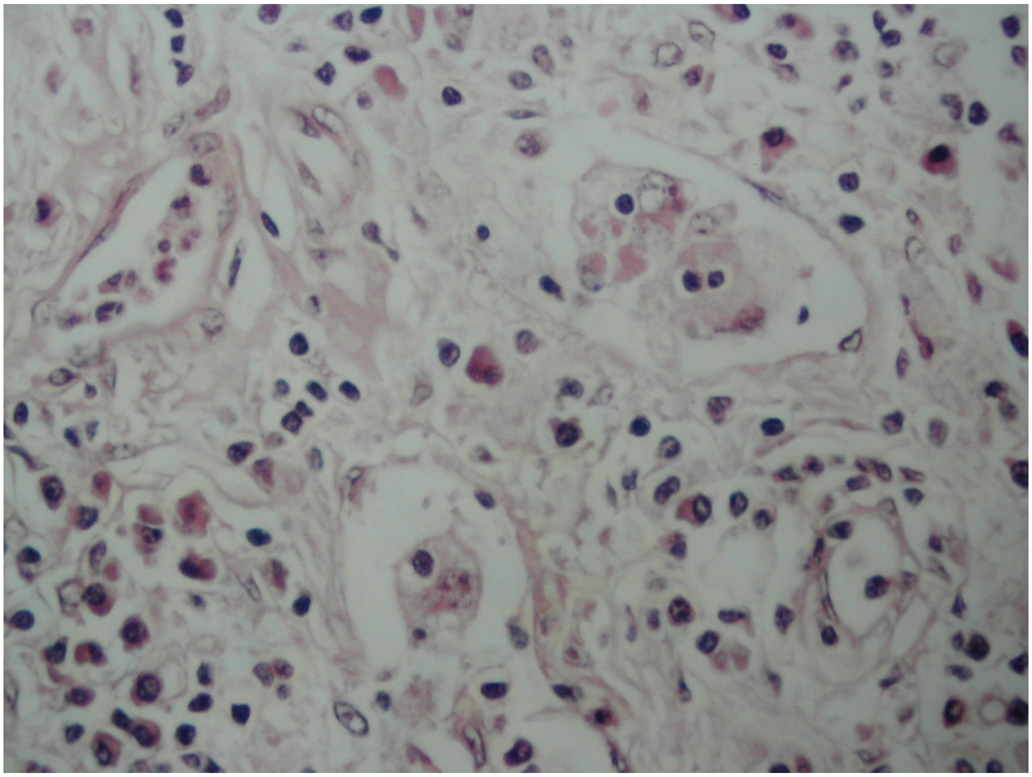

**FIGURE 2.** Pleural biopsy specimen: infiltrate with lymphoid cell clusters alternating with pale areas representing the histiocytic cell population (hematoxylin-eosin stain; magnitude ×40).

The natural history of SHLM is variable and typically reported to follow a benign course with spontaneous resolution within a period of several months to several years. It must be stressed that lower respiratory tract involvement in SHLM is unfavorable and carries a particularly grave prognosis, as happened in our case[4].

Treatment does not appear to be necessary in the majority of patients with SHLM since the disease is often self-limited and subject to spontaneous regression. The major indication for surgery other than biopsy is life- or function-threatening obstruction. Patients with progressive symptoms have been treated with chemotherapy, such as alkylating agents and corticosteroids. However, these treatments did not produce effective results[5].
